# The Mucosal Immune System and Its Regulation by Autophagy

**DOI:** 10.3389/fimmu.2016.00240

**Published:** 2016-06-22

**Authors:** Agnieszka M. Kabat, Johanna Pott, Kevin J. Maloy

**Affiliations:** ^1^Sir William Dunn School of Pathology, University of Oxford, Oxford, UK

**Keywords:** IBD, autophagy, ATG16L1, colitis, Treg cells, intestinal epithelial cells, metabolism, inflammasome

## Abstract

The gastrointestinal tract presents a unique challenge to the mucosal immune system, which has to constantly monitor the vast surface for the presence of pathogens, while at the same time maintaining tolerance to beneficial or innocuous antigens. In the intestinal mucosa, specialized innate and adaptive immune components participate in directing appropriate immune responses toward these diverse challenges. Recent studies provide compelling evidence that the process of autophagy influences several aspects of mucosal immune responses. Initially described as a “self-eating” survival pathway that enables nutrient recycling during starvation, autophagy has now been connected to multiple cellular responses, including several aspects of immunity. Initial links between autophagy and host immunity came from the observations that autophagy can target intracellular bacteria for degradation. However, subsequent studies indicated that autophagy plays a much broader role in immune responses, as it can impact antigen processing, thymic selection, lymphocyte homeostasis, and the regulation of immunoglobulin and cytokine secretion. In this review, we provide a comprehensive overview of mucosal immune cells and discuss how autophagy influences many aspects of their physiology and function. We focus on cell type-specific roles of autophagy in the gut, with a particular emphasis on the effects of autophagy on the intestinal T cell compartment. We also provide a perspective on how manipulation of autophagy may potentially be used to treat mucosal inflammatory disorders.

## Introduction

**Figure 1:**
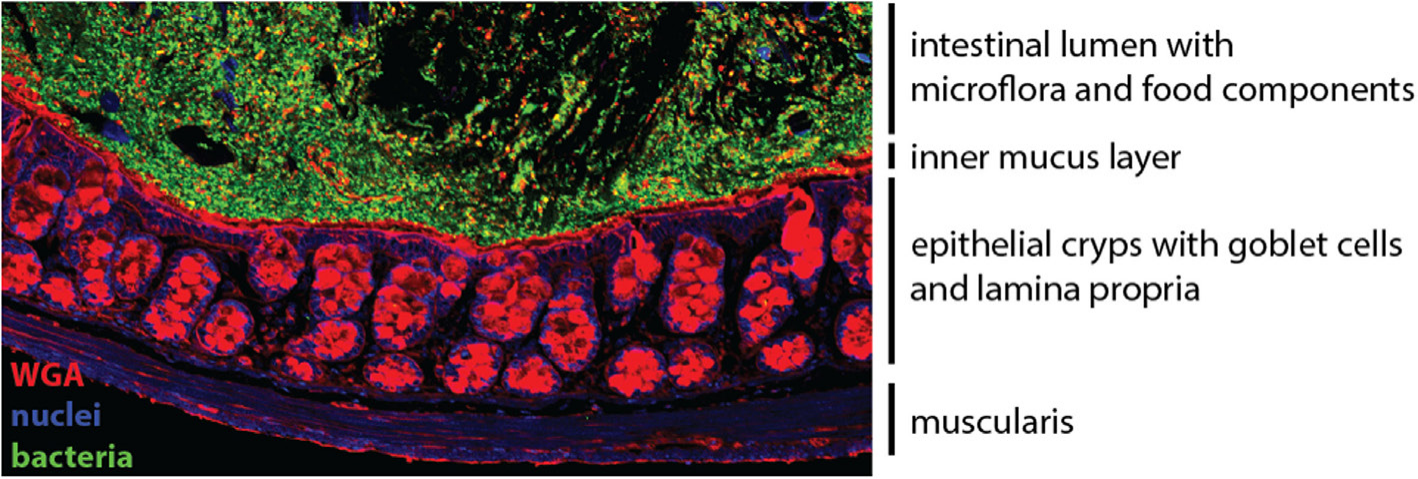
**The colonic mucosa**. The epithelial cell layer of the intestinal mucosa separates the luminal content harboring the microflora (green) from the underlying lamina propria. Specialized secretory epithelial cells, termed goblet cells, produce and secrete mucus to enforce the barrier (red). Cell nuclei are stained in blue.

The gastrointestinal tract contains a vast network of non-lymphoid and secondary lymphoid tissues that host numerous populations of leukocytes, many of which are intestine-specific subpopulations (1). The gut-associated lymphoid tissue (GALT) comprises the immune cells residing in the intestinal epithelium and lamina propria (LP) compartments, as well as various secondary lymphoid structures, including the mesenteric lymph nodes (mLNs), the Peyer’s patches (PP) of the small intestine, and isolated lymphoid follicles and cryptopatches that are distributed throughout the intestine (2). The intestinal mucosa, comprising the epithelium, the underlying LP, and the muscularis mucosa, is the site where majority of immunological processes occur (Figure 1).

The immune system has evolved to prevent invasion of the host by microbial species. However, the mammalian gastrointestinal tract is a preferential site for colonization of the host by commensals, a diverse community consisting of fungal, viral, and bacterial species. The presence of a commensal microbiota is vital for optimal digestion and nutrient acquisition, as well as resistance to pathogenic infection, but commensal microbes also contribute to the development, maturation, and activation of the host immune system by influencing both innate and adaptive immune responses (3). A mutualistic dialog between the microbiota and intestinal immune system maintains peaceful coexistence, through multiple mechanisms that we are just beginning to understand (3, 4). Besides microbial communities, the intestinal immune system constantly encounters a vast dietary antigenic load, and the induction of a state of immune unresponsiveness toward these antigens (oral tolerance) is key function of the mucosal immune system (5). Therefore, the intestinal immune system often employs different rules than the systemic immune system to ensure the right balance between tolerance and immunity is maintained. Disruption of this equilibrium can lead to the chronic immune-mediated pathologies of the gastrointestinal tract, such as inflammatory bowel disease (IBD) or food allergies (5, 6).

The term IBD describes a spectrum of chronic incurable inflammatory disorders affecting the gastrointestinal tract, but often with extra-intestinal manifestations, with the two most common forms being Crohn’s disease (CD) and ulcerative colitis (UC) (7). IBD is a complex multifactorial disease that emerges on a background of many genetic and environmental factors (6). In recent years, there has been a tremendous progress in understanding the genetics of IBD susceptibility, facilitated by technological progress that led to large-scale genome-wide association studies (GWAS), followed by meta-analysis and targeted genotype arrays (Immunochip) (8). Currently, 163 loci associated with IBD have been identified, which are far more than any other complex immunological disease to date (9). A considerable proportion of IBD-associated genes are involved in immune cell function, innate and adaptive immune responses, promotion of epithelial barrier integrity and bacterial handling (9). Among these, a single-nucleotide polymorphism (SNP) in *ATG16L1* was identified as a strongly associated risk locus for CD, suggesting for the first time a role for the macroautophagy (herein referred to as autophagy) pathway in IBD (10, 11). Importantly, it was recently shown that homozygous expression of the CD susceptibility variant T300A allele of *ATG16L1* results in defective autophagy during stress conditions (12, 13). Additionally, SNPs in several other autophagy-associated genes, including *IRGM, LRRK2, SMURF1*, and *NDP52*, have been linked to IBD susceptibility (9), strongly suggesting that it is the classical autophagy pathway that connects these genetic alterations to impaired intestinal homeostasis. However, the identification of susceptibility polymorphisms provides only correlative evidence for the involvement of specific genes or pathways, and an understanding of the functional consequences of the majority of genetic polymorphisms is still lacking. In the case of autophagy, however, we are beginning to understand how modulation of this pathway affects various aspects of mucosal immune cell physiology.

## The Autophagy Pathway

Degradation and recycling of cellular components is critical for all eukaryotic cells in order to maintain cellular homeostasis. The autophagy pathway degrades large cytoplasmic components, including organelles, long-lived proteins, and protein aggregates, as well as intracellular pathogens, by sequestering these constituents in double-membrane vesicles and delivering this cargo for lysosomal degradation (14). Autophagy is an evolutionary conserved process occurring throughout the eukaryotic phylogenetic tree, with the core autophagy machinery proteins showing great homology between yeast and mammalian cells (15, 16), and its essential physiological role is indicated by the observation that mice lacking essential autophagy genes are unable to survive the neonatal starvation period and die shortly after birth (17).

**Figure 2:**
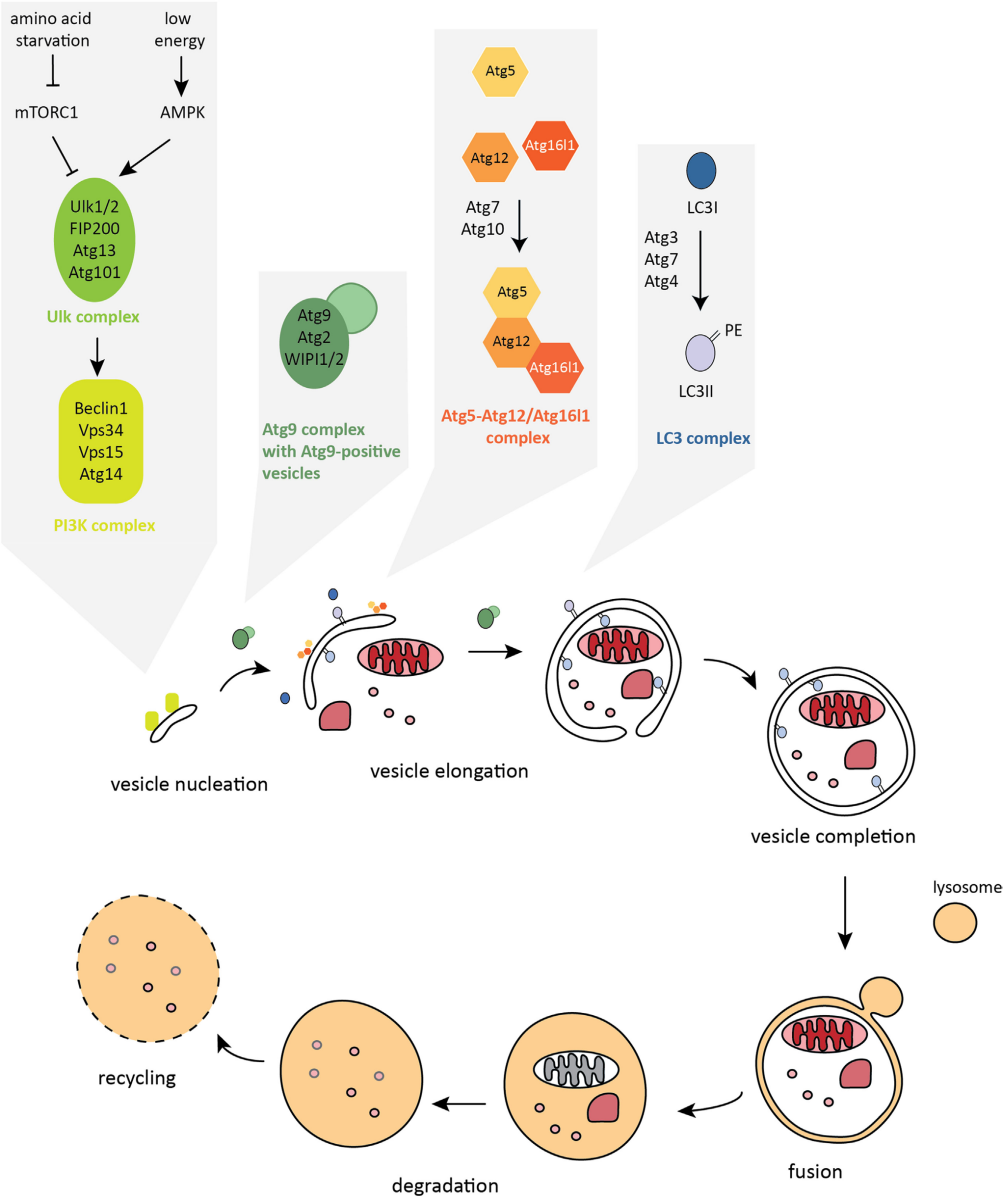
**The autophagy pathway**. Autophagosome formation is a stepwise process characterized by dynamic remodeling of cytoplasmic membranes. Proteins that control activation, elongation, and completion of an autophagosome are grouped into five functional complexes that are active at different stages of the autophagy pathway.

During autophagy, sequestration of the cytosolic cargo involves “*de novo*” formation of an isolation membrane that surrounds the cytosolic material to be degraded, forming an intermediate vesicle called an autophagosome. The autophagosome subsequently fuses with the lysosome leading to the formation of the digestive compartment – the autolysosome. Lysosomal enzymes degrade the content of the vesicle, which facilitates the permease-mediated release of the recycled molecules *via* the lysosomal membrane (16, 18, 19) (Figure 2). While autophagy is the primary cell response to the stress of nutrient deprivation, in recent years, more complex and cell type-specific functions have emerged, including roles in innate and adaptive immune responses (20, 21).

Although originally described as a non-selective pathway for bulk degradation, autophagy can also act as a highly selective process. While metabolic stress mainly triggers non-selective autophagy where a portion of the cytoplasm is targeted for degradation, intracellular pathogens or damaged organelles are targeted in a selective fashion. This is achieved by the use of cargo-specific autophagy adaptors. These adaptors are able to recognize ubiquitinated substrates and target them to the autophagosome, a process that also requires adaptor binding to the protein LC3 (microtubule-associated protein L chain 3) or γ-aminobutyric acid receptor-associated proteins (GABARAP), through a specific amino acid sequence called the LC3-interacting region (LIR) (22, 23). To date, there is evidence for the targeted sequestration and selective autophagy of a diverse array of cytosolic cargos, including aggregate-prone or misfolded proteins (aggrephagy) (22, 24–26), protein complexes in signaling cascades (27–29), peroxisomes (pexophagy) (30, 31), mitochondria (mitophagy) (32–38), surplus ER (reticulophagy) (39, 40), ribosomes (ribophagy) (40, 41), ferritin (ferritinophagy) (42, 43), bacteria and viruses (xenophagy) (21), lipid droplets (lipophagy) (44), and glycogen (glycophagy) (45, 46). The diversity of autophagy targets highlights the complex role of this pathway in regulating many aspects of cellular physiology during steady-state and in stress responses.

### Molecular Mechanisms of Autophagy

Formation of the double-membrane autophagosome structure is the key step in autophagy. There are nearly 40 autophagy-related (Atg) proteins that facilitate crucial steps of autophagosome formation and degradation (47). Autophagy is initiated by the formation of the isolation membrane, also called a phagophore, at the phagophore assembly site (PAS). The core autophagy machinery in mammalian cells can be categorized into five functional groups (Figure 2). The primary initiation complex, comprising unc-51-like kinase-1 or 2 (Ulk1/2) – Atg13 – FIP200 – Atg101, is reciprocally controlled by mechanistic target of rapamycin complex 1 (mTORC1) and AMP-activated protein kinase (AMPK). Activated Ulk1/2 complex translocates to the site of autophagosome formation and activates the second functional complex, the class III phosphatidylinositol 3-kinase (PI3K) complex, whose main components are vacuolar protein sorting 34 (Vps34), Beclin1, autophagy/beclin-1 regulator 1 (AMBRA1), and Atg14 (48–51). The PI3K complex mediates the nucleation step of phagophore formation. Once activated, this complex produces phosphatidylinositol-3-phosphate (PI3P) leading to recruitment of PI3P-binding effector proteins to the phagophore membrane, including WD-repeat domain phosphatidylinositide-interacting-1 or -2 (WIPI1/2) (52–54). The third complex consists of Atg9, the only known transmembrane Atg protein, and its cycling system, involving Atg2 and WIPI1/2, that shuttle among endosomes, autophagosomes, and the Golgi apparatus. Atg9-positive vesicles are thought to provide membrane for the growing autophagosome (55, 56). Once initiated, elongation of the isolation membrane requires the subsequent action of two ubiquitin-like (UBL) conjugation systems: the Atg5–Atg12/Atg16l1 complex, which is assembled through the action of Atg7 (E1-like enzyme) and Atg10 (E2-like enzyme) and then locates to the growing autophagosome membrane (57–61); and the LC3 complex, containing LC3A/B/C, GABARAP, GABARAPL1/2, Atg7, Atg3 (E2-like enzyme), and the cysteine protease Atg4, which cleaves LC3 at the C-terminus to expose glycine, allowing the conjugation of the membrane lipid phosphatidylethanolamine (PE) to the soluble form of LC3, named LC3-I, and subsequent incorporation of LC3-PE (also called LC3-II) into the inner and outer membranes of autophagosomes (57, 62–64). The Atg5–Atg12/Atg16l1 complex acts as an E3-like enzyme during LC3 lipidation (58, 65). LC3-II that is bound to the isolation membrane is thought to play not only a scaffolding role in membrane growth and is needed for autophagosome closure but is also important for the binding of autophagy adaptors and thus in mediating selective types of autophagy (66). The mature autophagosome migrates into close proximity to the lysosome through the action of the dynein motor complex and microtubules (67). Fusion with the lysosome requires the action of the HOPS complex (homotypic fusion and protein sorting) and SNARE proteins (soluble NSF attachment protein receptors) (68–71). Upon formation of the autolysosome compartment, lysosomal acidic hydrolases degrade the inner membrane and the luminal content. Ultimately, this provides building blocks for anabolic processes and fuel for ATP synthesis. Eventually, the autolysosome fissions to release lysosomes and autophagy are terminated (72).

### Cytoplasmic and Nuclear Regulation of Autophagy

Autophagy is modulated in response to adverse micronenvironmental conditions, including nutrient depletion, hypoxia, growth factor withdrawal, inflammatory cytokines, and infection. As such, a network of regulatory pathways governs its activity. For a long time, autophagy was believed to be predominantly regulated at the post-transcriptional level by signaling mediators. Recently, however, mechanisms of transcriptional, translational, and epigenetic regulation of the autophagy pathway have emerged. It is now thought that cytosolic regulation generally serves as a more rapid, short-term response, whereas transcriptional modulation provides long-term regulation, although some nuclear events can also have a rapid effect on autophagy (72–74).

The primary role of autophagy is to respond to cellular metabolic perturbations. Many signals that modulate autophagy levels do so by converging on the mechanistic target of rapamycin complex 1 (mTORC1). mTOR is a conserved serine/threonine kinase that integrates signals from various stimuli, including amino acids, growth factors, energy, glucose, and oxygen levels (75). The autophagy pathway is regulated by mTORC1 in several ways. In the presence of nutrients, including amino acids, mTORC1 is active and suppresses autophagy through inhibitory phosphorylation of Ulk1, Atg13, AMBRA, and Atg14 (51, 76–80). During starvation, mTORC1 is inhibited, which activates autophagy. Autophagy activity eventually leads to increased nutrient levels, which in turn reactivates mTORC1 to terminate autophagy. This reactivation of mTORC1 that occurs during prolonged starvation is critical for the restoration of lysosomal homeostasis after prolonged autophagy (81). Another key sensor that coordinates cellular metabolic responses is AMPK, which, next to mTOR, can be considered a signaling hub for autophagy modulation. AMPK is a serine/threonine kinase that senses decreased energy levels by detecting changes in the ATP:ADP:AMP ratio in the cytoplasm (82). In response to decreased intracellular ATP levels, AMPK initiates metabolic reprograming toward catabolic reactions, including stimulation of autophagy through direct phosphorylation of the Ulk1/2 (76, 83, 84). AMPK can also phosphorylate various components of the Vps34 complexes that do not contain proautophagic adaptors, leading to the inhibition of its non-autophagic functions in Golgi–endosome trafficking (85). In addition to direct interactions, AMPK can indirectly activate autophagy by inhibiting mTORC1 (86, 87). Importantly though, AMPK activity is not indispensable for autophagy induction, as starvation still induces autophagy in AMPK-null cells, suggesting a role for this kinase in fine-tuning autophagy modulation (76).

Transcriptional regulation is now appreciated to be one of the main regulatory mechanisms of autophagy. The transcription factor EB (TFEB) is a master regulator of lysosomal and autophagy gene expression (88). TFEB controls the gene network called coordinated lysosomal expression and regulation (CLEAR), which contains the majority of genes encoding lysosomal proteins (89). TFEB also regulates the expression of several genes encoding proteins belonging to the core autophagy machinery, including *Lc3, p62*, and *Atg9* (73, 88). Upon autophagy induction, TFEB is rapidly recruited from the cytosol to the nucleus, and this is at least partially mediated by inhibition of mTORC1. Active mTORC1 mediates the phosphorylation of TFEB, which results in its sequestration in the cytoplasm (90–92). Acting in opposition to TFEB, the DNA-binding protein zinc-finger protein with KRAB and SCAN domains 3 (ZKSCAN3) represses an extensive set of autophagy genes, including *Lc3* and *WIPI2* (93). During starvation, ZKSCAN3 accumulates in the cytoplasm, and its activity is inhibited. Thus, TFEB and ZKSCAN3 seem to provide a switch mechanism during starvation-induced autophagy (73). In summary, multiple intersecting pathways modulate autophagy on many levels, reflecting the importance of this complex homeostatic pathway in the cellular adaptations to environmental factors.

## Autophagy Has Diverse Roles in Cells of the Intestinal Mucosa

### Intestinal Epithelial Cells in Barrier Function and Immune Homeostasis

Intestinal epithelial cells (IECs) form a single cell layer separating the intestinal mucosa from the lumen. The primary function of these cells is nutrient absorption from the lumen; however, their interactions with the intestinal microbiota and host leukocytes strongly influence immune responses (94). The IEC monolayer is composed of several specialized cell types: stem cells, Paneth cells, goblet cells, neuroendocrine cells, and enteroabsorptive cells (95). Multipotent Lgr5^+^ stem cells are located at the bottom of the intestinal crypts and by division these cells give rise to either transient amplifying cells or stem cells. The transient amplifying cells rapidly proliferate and differentiate and thereby ensure the renewal of the epithelial layer every 4–5 days (95). Paneth cells that localize to the base of small intestinal crypts are specialized secretory cells that produce large amounts of antimicrobial molecules, including lysozyme, α-defensins, and Reg3γ (Reg3α in humans) (96). Antimicrobial peptides (AMP) play a crucial role not only in the defense against enteric pathogens but also in shaping the host microbiota, as mice lacking MMP7, an enzyme required for the maturation of α-defensins, exhibited significant changes in the microbiota composition (97). In addition, AMP have modulatory functions in chemotaxis, toll-like receptors (TLR) signaling, and wound healing (98). Paneth cells also participate in maintaining crypt stem cell activity through production of EGF, TGF-α, Wnt3, and the Notch ligand Dll4 (99). Goblet cells are another class of secretory cells that produce heavily glycosylated mucins which, after secretion to the lumen, form a mucus gel layer (94). This serves as a protective physical barrier and as a matrix loaded with secretory IgA (sIgA) and AMP, which fortify the mucosal barrier (4). Recent studies have suggested that mucus also has an additional role in promoting tolerogenic responses toward food and commensal antigens (100, 101). The observation that mice with defects in MUC2 production develop spontaneous colitis emphasizes that mucus is essential for intestinal homeostasis (102, 103). IECs are actively engaged in the dialog between the microbiota and the immune system. Sensing of bacterial metabolites and structural components by IECs fortifies barrier integrity and protects from pathogen invasion (104). For example, recent studies underlined the crucial role of inflammasome signaling in the epithelium in regulating microbiota composition and for protection against infectious colitis (105–107). In addition, the metabolite acetate, produced by commensal bacteria belonging to the genus *Bifidobacterium*, protected against mortality during enterohemorrhagic *Escherichia coli* infection by promoting anti-apoptotic responses in IECs (108). IECs also influence the recruitment, activation and differentiation of leukocytes by producing various other modulatory factors in response to commensal microbiota, including thymic stromal lymphopoietin (TSLP), TGF-β1, RA, IL-25, and IL-18 (4, 109).

### Autophagy Reinforces Barrier and Secretory Functions of IECs

**Figure 3:**
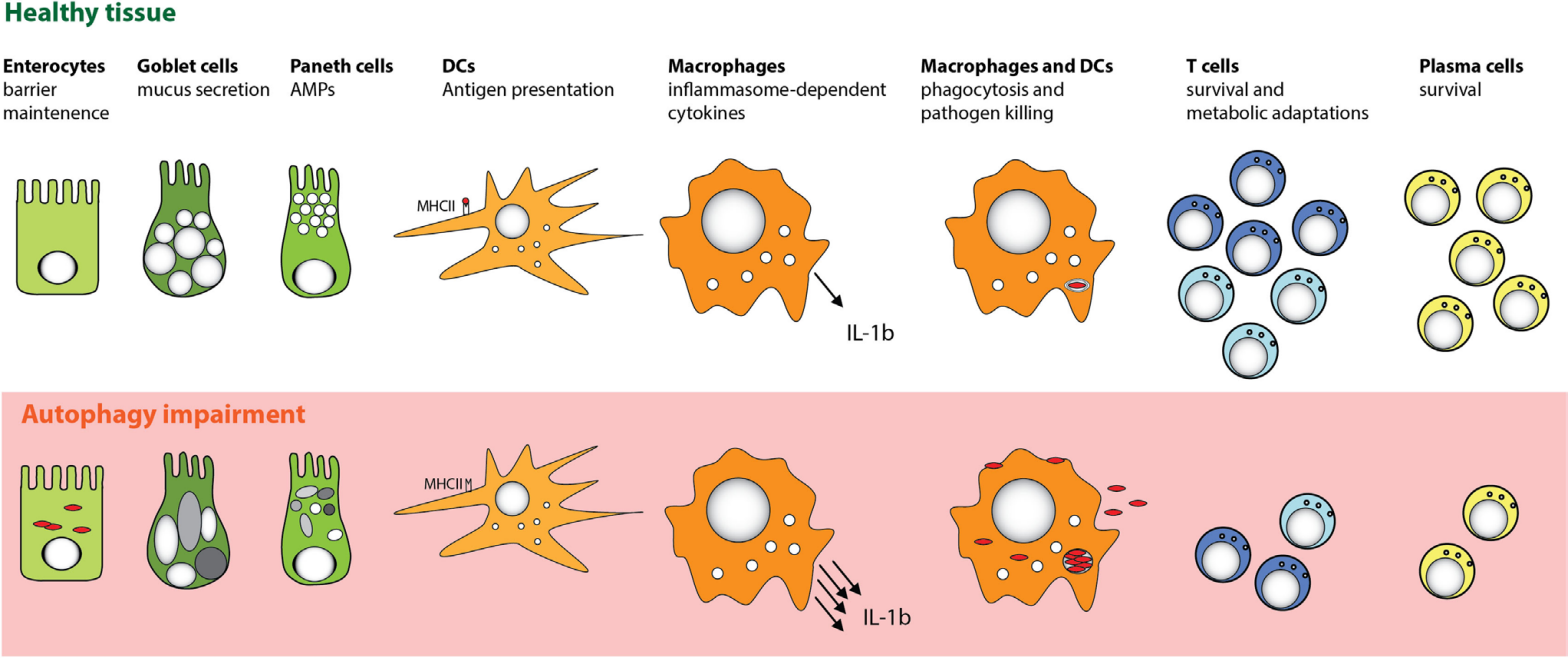
**Cell-specific functions of autophagy in the intestinal mucosa**. Autophagy pathway is essential for several key functions of distinct cell types that promote intestinal immune homeostasis. Perturbation in the autophagy pathway results in decreased antibacterial defense in IECs and MPs. Autophagy also facilitates secretory functions of Paneth cells and goblet cells, is involved in antigen presentation by DC, and limits proinflammatory cytokine production from MP. Furthermore, defects in autophagy pathway strongly compromise the survival of particular subsets of T cells and B cells.

The IEC monolayer is in close proximity to microbiota communities within the gastrointestinal tract and is an entry site for mucosal pathogens. Recent studies that assessed the impact of autophagy deficiency on bacterial handling by IECs found that autophagy was essential for protection against intracellular bacteria, including *Salmonella typhimurium* and *Shigella flexneri*, by acting to limit bacterial replication and subsequent dissemination to other tissues (13, 110–112) (Figure 3). Furthermore, a recent study showed that autophagy induction reduced tight junction permeability in IECs by targeting the pore forming protein claudin-2 for degradation (113), highlighting an additional mechanism through which autophagy may enhance barrier function.

Many studies of autophagy in IECs have concentrated on the functional role of ATG16L1, and in trying to understand how CD-associated polymorphisms in *ATG16L1* may impact on epithelial homeostasis. It has been reported that Paneth cells from patients with CD homozygous for the *ATG16L1* T300A variant allele, or from mice with a hypomorphic mutation of *Atg16l1*, exhibit abnormal granule structure and reduced AMP secretion (114, 115) (Figure 3). However, *Atg16l1* hypomorphic mice did not exhibit signs of spontaneous intestinal inflammation, although they showed increased susceptibility to DSS-induced colitis. This increased susceptibility of *Atg16l1* hypomorphic mice, as well as their Paneth cell abnormalities, was only observed when the mice harbored a commensal microbiota that contained a persistent enteric norovirus (MNV) (114, 115). These studies suggested that decreased autophagy levels drive Paneth cell abnormalities only when additional triggering factors are present. A recent study showed that ER stress could also be such a trigger (116). Analyses of mice with an IEC-specific deletion of the unfolded protein response (UPR) response element *Xbp1* demonstrated that defects in the UPR pathway in Paneth cells were partially compensated by increased autophagy. However, when autophagy was also impaired, through IEC-specific deletion of *Atg16l1* or *Atg7*, ER stress could not be resolved and this double defect led to the development of severe intestinal inflammation (116). Interestingly, Paneth cells from patients with the *ATG16L1 T300A* variant allele showed increased ER stress markers (117).

Consistent with the notion that manifestation of defective autophagy in Paneth cells could depend on additional environmental or genetic factors, two recent studies reported contradictory results when mice with a “knock-in” of the risk-associated *Atg16l1* gene variant (T316A) were analyzed. Murthy et al. reported no changes in the morphology of Paneth cells in the knock-in mice (12), whereas Lassen et al. observed spontaneous Paneth cell abnormalities (13). One possible explanation for these differences could be the distinct microbiota composition of mice housed in different facilities. Beyond Paneth cells, autophagy could also play an important functional role in other secretory IEC types, as *Atg5*-deficient colonic goblet cells were also reported to show impaired granule formation (118). Taken together, these studies indicate that autophagy plays an important role in fortifying intestinal epithelial barrier function by enhancing resistance to intracellular bacteria and by regulating the functions of secretory IECs (Figure 3).

### Mononuclear Phagocytes Regulate Intestinal Immune Homeostasis

Mucosal mononuclear phagocytes (MPs) comprise dendritic cells (DCs) and macrophages. These cells are key players in intestinal homeostasis, as they provide a crucial link between innate and adaptive immunity, and in maintaining functional compartmentalization of the systemic and mucosal immune system. Intestinal MPs are a heterogeneous population; expression of CX_3_C chemokine receptor 1 (CX_3_CR1) and CD103 (αE integrin) can be used to identify two major intestinal MP populations, which appear to promote intestinal tolerance in different ways (119, 120). Under homeostatic conditions, CD103^+^ DCs acquire intestinal antigens and migrate from the intestinal LP to the mLNs, where they initiate T cell responses, promoting intestinal tropism through induction of homing receptors CCR9 and α4β7, and preferentially inducing tolerogenic Treg cells through production of TGF-β_1_ and RA (121–123). The ability to convert latent TGF-β1 into its active form is important for this tolerogenic CD103^+^ DC function (124, 125). Additionally, this CD103^+^ DC subset also acts on B cells, promoting differentiation of naive B cells into IgA^+^ plasma cells within the intestinal LP (126). CD103^+^ DCs have also been identified as a crucial subset in promoting oral tolerance against food antigens (5), although CX_3_CR1^+^ MPs may augment Treg cell induction by CD103^+^ DCs through the transfer of soluble food antigens *via* gap junctions (127). CX_3_CR1^+^ MPs (a population comprising both DCs and macrophages) sample luminal contents through extended dendrites but appear to be non-migratory and have poor abilities for naive T cell priming (128–130). However, CX_3_CR1^+^ MPs may be involved in the secondary expansion of Tregs in the LP that had been primed initially in gut-draining lymph nodes (131).

During infection or inflammation, intestinal MPs adopt a different phenotype, characterized by the production of proinflammatory cytokines and chemokines that coordinate host protective immune responses, but excessive activation of mucosal myeloid cells has also been associated with chronic inflammatory conditions (132–134). Commensal bacteria are able to directly modulate intestinal MP functions to regulate effector T cell responses in the LP. For instance, Atarashi et al. showed that ATP produced by commensal bacteria activates CX_3_CR1^+^ MPs and leads to the induction of Th17 cells (135), and microbiota-derived signals were shown to induce IL-1β production from mucosal MPs that is essential for the induction of Th17 cells in the steady-state gut (136).

### Autophagy Regulates Proinflammatory Signaling in Mononuclear Phagocytes

Antigen presentation by intestinal MP is crucial in orchestrating protective and tolerogenic responses in the mucosa. The role of autophagy in major histocompatibility complex class II (MHC II) antigen presentation is well documented; autophagy can enhance MHC II expression on MPs and is directly engaged in delivering cytoplasmic antigens, including bacterial and viral antigens, into MHC II compartments (137–140) (Figure 3). There is also evidence that autophagy may contribute to processing of viral antigens for MHC I presentation (141, 142).

As professional phagocytic cells, DCs and macrophages are particularly well equipped to handle bacteria, and autophagy is now appreciated to play an important role in intracellular bacterial killing (Figure 3). Autophagy can be activated in MPs by pattern recognition receptor (PRR) triggering, including activation of TLR (143–147) or NOD-like receptors (NLR) (112, 148). In particular, activation of DCs and macrophages with the NOD2 ligand muramyl di-peptide (MDP) induces autophagy and thereby enhanced bacterial killing, and this activation is reduced in DCs with the *ATG16L1* T300A allele variant (112, 148). Activation of NOD1 and NOD2 in the cytoplasm directs the autophagy machinery by recruiting ATG16L1 to the site of bacterial entry, although this interaction was not affected in cells with homozygous expression of the T300A variant (112). Of note, autophagy induction downstream of PRRs is not limited to myeloid cells, as autophagy is also activated in epithelial cells in response to NOD1 and NOD2 triggering (112), and bacterial outer membrane vesicles (OMV) were shown to selectively activate NOD1-dependent autophagy in an epithelial cell line (149).

Sequestration of cytosolic bacteria by xenophagy requires the coordinated action of specialized autophagy adaptors that recognize ubiquitin-tagged or galectin-tagged pathogens for degradation (150). The importance of autophagy in defense against cytosolic pathogens is highlighted by the fact that several pathogens have developed sophisticated adaptations to inhibit specific stages of autophagy (21, 151) and others even hijack the pathway for their own propagation (152). An essential role for autophagy in intestinal pathogen handling in DCs and macrophages has been observed in *Salmonella, Shigella*, and *Listeria* infections (153, 154).

**Figure 4:**
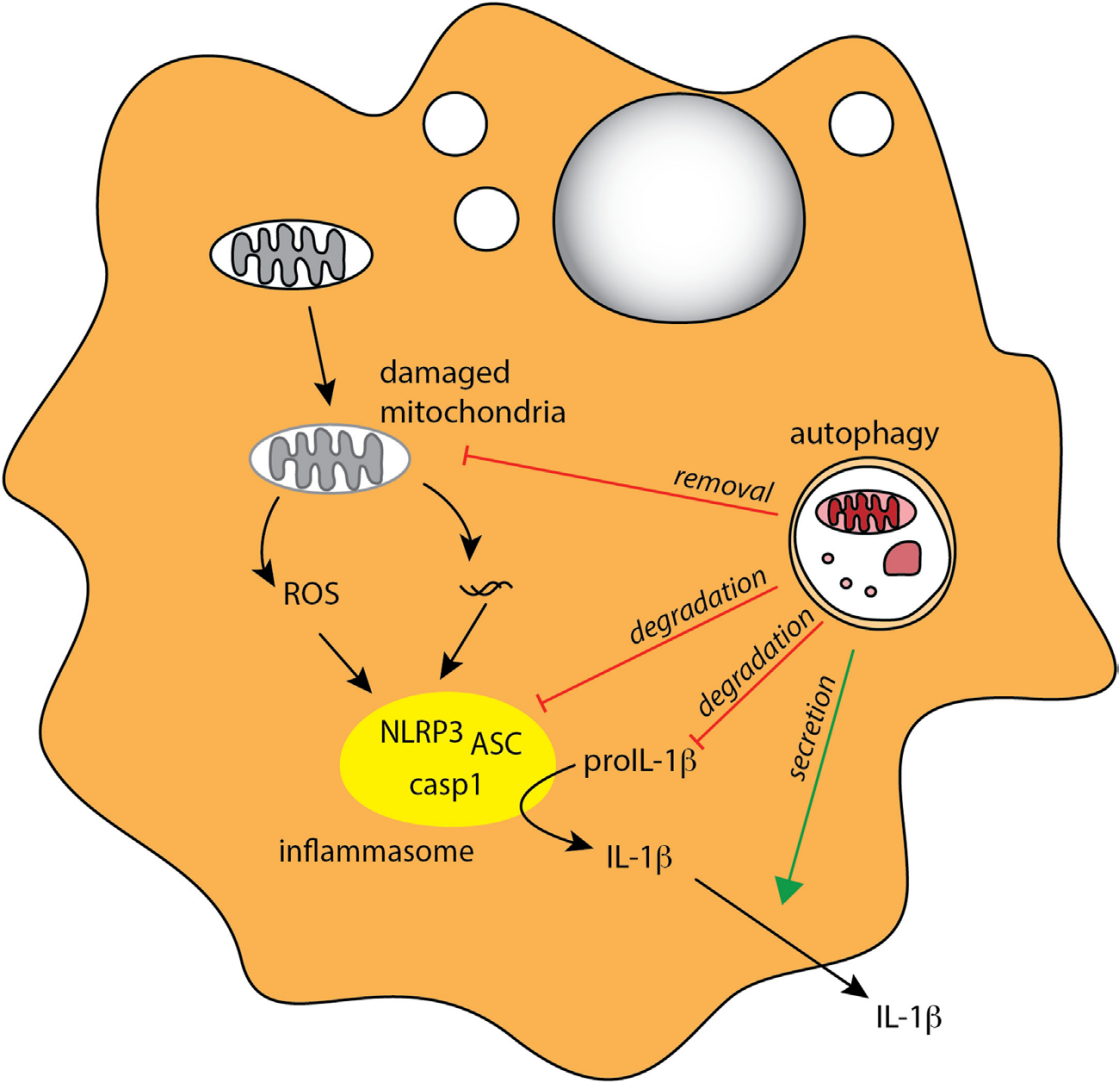
**Autophagy regulates inflammasome-associated cytokine responses**. Autophagy negatively regulates production of proinflammatory IL-1β in MPs through several mechanisms, indirectly through degradation of damaged mitochondria (mitophagy) to limit ROS production, and directly through degradation of inflammasome complexes and pro-IL-1β. Autophagy may also positively regulate IL-1β by mediating its unconventional extracellular secretion.

In addition to PRR triggering, inflammatory cytokines can also influence autophagy in MPs. For instance, autophagy is induced by IFN-γ and other Th1 type cytokines that are secreted during bacterial infection, while Th2 type cytokines inhibit autophagy (21). Conversely, autophagy can also influence cytokine signaling in myeloid cells. In particular, autophagy was shown to downregulate secretion of the inflammasome-associated cytokines IL-1β and IL-18 by murine and human macrophages (155–157) (Figure 3). In addition, IL-1β produced by activated autophagy-deficient macrophages can enhance their secretion of IL-23, thereby further potentiating inflammatory responses (157). The link between autophagy defects and excessive inflammasome activation was related to reactive oxygen species (ROS) production in response to mitochondrial stress (158–160), as well as defects in targeting of assembled inflammasomes, or components of the inflammasome pathway, for autophagosomal degradation (27, 161) (Figure 4). Recently, NFκB signaling was linked to autophagy-mediated silencing of inflammasomes in macrophages, as NFκB promoted the expression of the adaptor p62 that was needed for the removal of damaged mitochondria (162). In the absence of p62, signals from damaged mitochondria enhanced NLRP3 inflammasome activation (162). Consistent with these findings that autophagy functions as a key regulator of inflammasomes, in bone marrow chimeric mice with an Atg16l1-deficient hematopoietic compartment, increased production of inflammasome-dependent cytokines was associated with increased susceptibility to DSS-induced colitis (155). Furthermore, a recent study suggested that protective autophagy in MPs might be triggered through the activation of GCN2, a nutrient deprivation sensor kinase that was previously shown to facilitate antigen presentation by DCs through autophagy induction (163, 164). GCN2 promoted autophagy in intestinal MP during amino acid starvation or acute inflammation and this acted to limit ROS production and consequent inflammasome activation and thus had a protective effect on DSS colitis (164).

Although autophagy appears to negatively regulate inflammasome pathway at several stages, there is some evidence that, conversely, it might contribute to the secretion of IL-1β and IL-18 under some circumstances (165–167). IL-1β and IL-18 do not contain signal peptide and thus cannot access the classical secretory pathway; however, the mechanism of their unconventional secretion remains debatable. For instance, starvation-induced autophagy leads to increased IL-1β and IL-18 secretion in macrophages following inflammasome triggering, and this response was partially autophagy dependent (165). More recently, Zhang et al. proposed that autophagy-mediated secretion of IL-1β is mediated through sequestration of a fraction of this cytokine in the space between the membranes of the autophagosome, where it is protected from degradation (167).

Overall, these studies indicate that autophagy can both negatively and positively regulate inflammasome signaling (Figure 4) and suggest that this may depend on the timing and context of autophagy and inflammasome induction. It will now be important to fully understand what immunological clues dictate the location, and thus the fate of IL-1β and IL-18 within autophagosomes, and to investigate whether the tissue location of the MPs has an influence on how autophagy regulates inflammasome signaling.

Besides the crosstalk between autophagy and inflammasome signaling, autophagy also regulates type I IFN responses. In plasmacytoid DCs, autophagy was essential for IFN-α production in response to viral dsRNA, and this was attributed to the role of autophagy in delivering viral replication intermediates to endosomal TLR7 (147). Conversely, in some instances, autophagy appears to negatively regulate virus-sensing pathways by limiting signaling through RIG-1-like receptors (RLR) (168, 169). Additionally, autophagy was shown to enhance NFκB signaling in F4/80^hi^ macrophages by selective degradation of the negative regulator A20 and this contributed to enhanced protection against *Candida albicans* infection (170). This is interesting, as autophagy was previously shown to limit NFκB signaling in intestinal epithelial cells (171) and activated T cells through selective degradation of Bcl-10 (29), suggesting that the impact of autophagy on NFκB activation could be cell type specific or context dependent. Additionally, it was recently demonstrated that autophagy-deficient macrophages show increased secretion of macrophage migration inhibitory factor (MIF) (172), a pleiotropic proinflammatory cytokine implicated in the pathology of IBD (173, 174), and that this increase was dependent on the mitochondrial ROS (172). Taken together, these studies indicate that the autophagy pathway intersects with other pathogen sensing and cellular stress responses to promote immune defense in MP populations. However, detailed analysis of the role of autophagy in the intestinal subsets of DCs and macrophages is lacking. Since mucosal MPs show some unique features, including hyporesponsiveness to PRR stimulation (134), it will be important to investigate the potential contribution of autophagy to these specific intestinal adaptations.

### Antibody Production and Secretion in the Intestine

B cells are abundant within the GALT. Intestinal B cell development shows some unique features as LP-resident B cells appear to undergo V(D)J recombination and B cell receptor (BCR) editing (175). Early B cell development in the gut was promoted by commensals, suggesting involvement of microbiota-derived antigens in driving BCR editing (175), which might have implications for immunoglobulin diversification at mucosal sites and for tolerance against commensal antigens. Intestinal plasma cells can also acquire unique “innate like” properties that are dependent on microbiota stimulation, as IgA^+^ plasma cells can secrete TNFα and inducible nitric oxide synthase (iNOS) (176).

It is estimated that around 80% of the total antibody production takes place in the intestinal mucosa, making the gut the largest antibody-producing organ of the body (177). IgA constitutes the major antibody isotype produced and sIgA is the most abundant immunoglobulin in mucosal secretions (178). sIgA is a dimeric antibody that binds to the polymeric Ig receptor (pIgR) on the basolateral surface of IECs and is subsequently translocated across the epithelium and released into the lumen, where it interacts with various intestinal antigens, including self, dietary, and commensal antigens. This limits the access of commensal bacteria and soluble antigens to the intestinal epithelium and LP, and it appears that bacteria with higher potential to elicit local inflammatory responses can be distinguished on the basis of their high IgA coating (179, 180). Class switch recombination to IgA occurs mainly in the mLNs and PP through both T cell-dependent and T cell-independent mechanisms, and studies in GF mice established that commensal microbiota is strong inducers of IgA production (178, 179, 181). In the T cell-independent pathway, microbiota induces production of IgA through the modulation of IECs and MPs, which in turn secrete BAFF (B cell-activating factor), APRIL (a proliferation-inducing ligand), and TGF-β1, cytokines that promote IgA switching (178). Production of IgA in response to microbiota appears very flexible, as IgA specificity can rapidly change and adapt in response to alterations in microbiota composition (182, 183).

Pentameric IgM antibodies are also actively secreted into the intestinal lumen *via* the pIgR, have a similar function in shielding IECs from antigenic exposure, and are particularly important in newborns (177). There is also evidence that active transport across the epithelial layer takes place for IgG and IgE. In case of IgG antibodies, the neonatal Fc receptor (FcRn) is responsible for transcytosis across the IECs, and moreover, this transport is bidirectional, as IgG are able to bind antigen in the lumen and immune complexes may then be retrieved and released into LP, where they are proposed to provide antigen for DC sampling (184, 185). Mucosal sites, including the intestine, support isotope switching to IgE and indeed IgE is relatively abundant in the intestine (186, 187), with increased production being described in patients with food allergies (188, 189). IgE transcytosis across the intestinal epithelial barrier involves the low-affinity IgE-specific receptor CD23/FcRII, and, similar to IgG, also appears to be bidirectional, potentially resulting in antigen retrieval from the lumen to the intestinal LP (190–192). Active transport of IgE across the IECs to the lumen might have a particularly important role during helminth infections, as the concentration of IgE in the lumen after parasitic infection rapidly increases (193). However, the transport of antigen complexes from the lumen is thought to facilitate the rapid intestinal physiological changes that occur during allergic reactions to food antigens (187). Recent data demonstrated that IgE in the gut acts beyond driving immediate hypersensitivity reactions and mediates long-lasting immunomodulatory functions by enhancing the induction of proallergic Th2 cells and inhibiting Treg cell induction (194).

### Autophagy Regulates Plasma Cell Responses

In contrast to what has been described for T lymphocytes (see below), autophagy seems largely dispensable for the development and maintenance of mature B lymphocytes in the periphery. Studies using mice with B cell-specific deletion of Atg5 or Atg7 (Cre expressed under the control of the CD19 promoter: Atg5^ΔCD19^ or Atg7^ΔCD19^ mice) indicated that the numbers of mature B cells and the ratios of marginal-zone B cells to follicular B cells are not affected when autophagy is lacking (195–197). Interestingly, while B-2 and B-1b populations were not affected by Atg5 or Atg7 deficiency, the B-1a B cell population in the peritoneal cavity was markedly reduced. It remains unclear why the development of this population of peripheral B-1a cells is uniquely sensitive to autophagy deficiency (195–197).

However, studies on antibody responses, plasma cell and memory B cell formation provided evidence of a role for autophagy in regulating these processes (Figure 3). After examining the capacity of autophagy-deficient B cells to produce immunoglobulins, Conway et al. reported decreased primary antibody responses to antigen immunization or following infection with *Heligmosomoides polygyrus*, along with defective plasma cell differentiation (196). However, in contrast, a recent report observed no defects in primary antibody responses from autophagy-deficient B cells after antigen immunization (197). Moreover, an extensive analysis of autophagy-deficient plasma cells revealed that although autophagy did not affect differentiation or proliferation of plasma cells, it was necessary to promote their long-term survival (195). Surprisingly, immunoglobulin production was, in fact, increased in Atg5-deficient plasma cells, a phenomenon attributed to a dysregulated ER stress pathway. Signs of elevated ER stress were observed in autophagy-deficient plasma cells, leading to increased Blimp-1 expression, which in turn resulted in increased IgH expression and immunoglobulin production (195). However, after *in vivo* challenge with a pneumococcal vaccine, antibody levels were reduced, as a result of decreased survival of autophagy-deficient plasma cells (195). As the choice of the antigen and adjuvant for immunization had a significant impact on the *in vivo* antibody responses in Atg5^ΔCD19^ mice, it is plausible that some of the discrepant observations were due to the different immunization regimes (195). It is noteworthy that a requirement for autophagy appears to be shared by distinct types of professional secretory cells with high protein synthesis, as Paneth cells, goblet cells, and plasma cells are particularly sensitive to perturbations in autophagy and ER stress pathways.

Recent studies on B cell responses after influenza virus infection showed that although autophagy was dispensable for initial memory B cell formation, the survival of memory B cells and secondary antibody responses after re-challenge were heavily dependent on autophagy, and this defect could be partially rescued by treatment with a ROS scavenger (197, 198). These results parallel studies on memory CD8^+^ T cell responses during viral infections where autophagy was also implicated in the late stages of memory cell formation (199, 200).

Overall, it appears that autophagy, while largely dispensable for mature B lymphocyte development, is necessary to maintain secondary, long-lasting antibody responses. However, one study reported decreased numbers of B cells in the intestinal LP and PP in Atg5^ΔCD19^ mice, indicating that intestinal B cells might have a higher dependence on autophagy compared with other peripheral B cells, although this has not been further investigated (196). Taking into account the marked differences displayed by the intestinal B cell compartment and the high constitutive demand for local antibody production in the intestine, it would be of interest to address the potential role of autophagy in homeostasis of mucosal B cells and plasma cells.

## Autophagy Regulates T Cell Responses on Several Levels

### Autophagy Affects Thymic Selection of T Cells

Generation of T cells occurs in the thymus and requires continuous trafficking of bone marrow-derived lymphoid progenitor cells (201). Thymic selection acts to generate T cells that have self-MHC restricted TCR and do not display pathological self-reactivity. Thymocytes with the capacity of interacting with self-peptides presented on MHC I and MHC II molecules expressed on thymic cortical epithelial cells (cTEC) are positively selected toward the CD8 and CD4 lineages, respectively, whereas thymocytes that fail to proceed through positive selection die by neglect (202). Negative selection then ensures deletion of thymocytes with potentially pathogenic specificity. During negative selection, thymocytes cease TCR rearrangement and migrate to the medulla where they interact with peptides expressed by thymic medullary epithelial cells (mTEC) and thymic antigen-presenting cells (APCs) (203). Intermediate affinity to self-peptide–MHC complexes promotes survival, whereas high affinity leads to the removal of such self-reactive clones by induction of apoptosis (clonal deletion).

Interestingly, autophagy has been implicated in shaping the thymic repertoire. Presentation of certain self-antigens on cTEC was promoted by autophagy, implying a role in positive selection (204). During negative selection, it appears that autophagy in mTEC is dispensable for abundant antigens, as it can be compensated by presentation by thymic APCs, but may be more important when antigen is present at lower doses (205). However, the physiological relevance of autophagy-associated antigen presentation during negative selection remains controversial, as autoimmunity development depended on the experimental model used; athymic mice which received an *Atg5*-deficient thymus developed autoimmunity (204), but this was not the case in mice where *Atg7* was selectively deleted in thymic epithelium using a Cre-loxP approach (206). It is worth mentioning that several unconventional mucosal T cell subsets, such as invariant natural killer T (iNKT) cells, and CD8αα intraepithelial lymphocytes (IELs) undergo alternative thymic selection (207), and the impact of autophagy in the thymic epithelium on their selection is yet to be investigated.

### The Intestinal Mucosa Has a Unique T Cell Composition

The intestinal LP and epithelium together contain the largest population of T lymphocytes in the body (208). Gut T cells are highly heterogeneous, and many populations are unique to the mucosa. T cells found at the basement membrane between enterocytes are classified as IELs, which are particularly abundant in the small intestine. Two major subtypes can be distinguished in mice: conventional IELs, which express CD4 or the CD8αβ heterodimer as well as an αβTCR, and unconventional IELs, which express the CD8αα homodimer and either a γδTCR or an αβTCR (208). Overall, γδ T cells constitute a large proportion of IELs (approximately 60%), while CD4^+^ T cells are underrepresented (209). Intestinal IELs not only regulate epithelial growth and homeostasis, for example, through secretion of TGF-β1 (209) but are also essential in protection against pathogens, as γδ T cells are an important source of IL-17A (210).

The intestinal LP harbors a significant population of CD4^+^ T cells, which predominantly express TCRαβ. Conventional CD8^+^ T cells are also present, although in lower frequencies, and these give rise to effector cytotoxic T lymphocytes (CTLs) that combat intracellular pathogens (211–213). In addition, small subsets of T cells that express an invariant TCR are also present in the intestinal mucosa (208). These subsets include mucosal-associated invariant T (MAIT) cells and iNKT cells. MAIT cells express a semi-invariant TCR that recognizes bacteria-derived vitamin B metabolites presented by the MHC class I-related protein (MR1) (214). MAIT cells contribute to protection against enteric bacteria, as they rapidly produce cytokines and exert cytolytic activity upon activation (214). iNKT cells, which in mice constitute approximately 0.5% of small intestinal LP lymphocytes, express an invariant form of the TCRαβ that is able to recognize lipid antigens presented by the CD1d molecule (215). As well as cytolytic activity, iNKT cells can also rapidly produce a spectrum of effector cytokines at an early stage in immune responses, including IFN-γ, IL-4, IL-10, IL-13, and IL-17A, allowing these cells to participate in a range of immune responses, including antimicrobial defense (215).

Commensal microbiota modulates the function of IELs for example, GF mice (or mice treated with antibiotics) have reduced numbers IL-17 producing γδ T cells (216). In addition, the antimicrobial response of γδ T cells can be triggered by a distinct subset of commensal bacteria following penetration of the epithelial barrier (217). The influence of commensals also extends to iNKT cells, as exposure to commensal microbiota during the neonatal period limits the accumulation of iNKT cells at mucosal sites, which otherwise can have detrimental effects in adult animals, such as increased susceptibility to asthma (218). Mechanistically, the inhibition of colonic iNKT cell development by commensal microbiota is at least in part mediated by the interaction of iNKT cells with commensal-derived inhibitory sphingolipids (219).

Naive T cells are maintained in a metabolic state that favors energy production over biosynthesis and rely on mitochondrial oxidative pathways for maximal energy generation, fueled predominantly by lipid and amino acid oxidation (220). Within the intestinal mucosa, the majority of the T cells display an activated/memory phenotype (211). Activation of T cells initiates multiple changes in their transcriptional and translational program, which go hand in hand with dynamic metabolic changes, matching bioenergetic and biosynthetic demands. Activation of T cells drives a rapid proliferative response, which drastically increases the demand for energy and building blocks for biosynthesis (220). During the initial growth phase, lipid oxidation is downregulated, and glycolytic, pentose phosphate, and glutaminolytic pathways increase (221). This initial metabolic shift is orchestrated by the transcription factors c-Myc, hypoxia-inducible factor-1α (HIF-1α), and the nuclear receptor estrogen-related receptor α (ERRα), leading to an increase in amino acids, nucleic acids, and lipid synthesis (222–224). Toward the end of an immune response, a small proportion of the T cells differentiate into memory T cells and revert back to lipid oxidation, maintaining increased capacity for efficient energy generation (224).

Memory T cells are formed from both CD4^+^ and CD8^+^ effector T cells, and it is now appreciated that this long-term immunity is provided by several distinct subsets of memory cells that can be distinguish, based on their location and effector functions, into central memory T cells, effector memory T cells, and tissue-resident memory T cells (T_RM_) (225). Mucosal tissues are enriched in T_RM_; in contrast to other memory cells T_RM_ do not re-enter the circulation and are retained in the mucosa where they provide a rapid protection during a secondary local infection (226).

### T Helper Cell Populations in the Gut

Upon activation, CD4^+^ T cells differentiate into subsets of T helper cells, which have traditionally been classified according to the expression of the lineage-specifying transcription factors (so-called master transcriptional regulators) and effector cytokine profiles. As such, Th1, Th2, Th17, and T follicular helper (Tfh) effector cells can be distinguished. Metabolic signals and the surrounding cytokine milieu greatly affect the differentiation process (227). As CD4^+^ T cells begin to proliferate and differentiate, metabolic programs support the commitment into separate lineages, with major roles for mTOR and AMPK in tailoring the metabolic adaptations of particular CD4^+^ T cell subsets (228, 229). Although this paradigm provides a useful framework for defining different functional CD4^+^ T cell responses, recent data suggest that it oversimplifies the dynamic interactions within the transcriptional network that orchestrates CD4^+^ T cell differentiation, which in turn provides a degree of functional and phenotypic plasticity within CD4^+^ T cell subsets (230, 231). Nevertheless, the functional specialization of CD4^+^ T cells generates effective immune responses that are tailored to meet particular infectious or inflammatory insults.

Th1 cells are characterized by production of the signature cytokine IFN-γ, but can also secrete TNF-α, GM-CSF, and lymphotoxin, and they are considered good producers of IL-2 (227). T-bet (T-box expressed in T cells) is the master transcription factor that orchestrates Th1 cell development (232). Metabolic sensors that favor differentiation toward Th1 lineage include signaling through mTORC1, which results in a strong engagement of glycolysis (220). Through the production of IFN-γ, Th1 cells are potent activators of macrophages and thus play a major role in the defense against intracellular pathogens such as *Leishmania* (233) and *Toxoplasma* (234, 235). However, aberrant Th1 responses have been implicated in several chronic inflammatory disorders, including type I diabetes (236), multiple sclerosis (237), and IBD (238).

Th2 cells are characterized by the production of IL-4, IL-5, IL-9, IL-13, and amphiregulin. IL-4 plays a crucial role in directing Th2 polarization (239). Other factors that promote Th2 differentiation include the alarmins TSLP, IL-33, and IL-25, which are released by epithelial cells in response to tissue injury and prime DCs and basophils to promote Th2 responses (240, 241). Th2 cell lineage commitment is orchestrated by the transcription factor Gata3, which is induced in response to IL-4 driven activation of STAT6 (242, 243). Although mTORC1 signaling is needed for Th2 cell lineage specification, Th2 cells are considered to be more reliant on mTORC2 in comparison to other T helper subsets (244, 245). Th2 responses are important in promoting tissue repair pathways (246), defense against large helminth parasites (247), resistance against toxins and venoms (248), and regulation of glucose homeostasis, adiposity, and thermogenesis (249, 250). However, the host protective functions of type 2 immunity are mirrored by detrimental effects when their activation is persistent or dysregulated. As such, type 2 responses can induce fibrosis, promote allergic diseases, including asthma and food allergies, and antagonize anti-tumor defense (241).

T follicular helper cells are becoming recognized as a separated lineage of CD4^+^ T cells that specialize in the provision of help for B cell responses. Tfh assistance is essential to induce maturation, isotype switching, and terminal differentiation of B cells (251), events that occur mainly within germinal centers (GC). As such, Tfh cells are essential for the production of most types of antibodies, although their role in IgE responses remains unclear (252). The transcriptional repressor B cell lymphoma 6 (Bcl-6) orchestrates Tfh lineage commitment and is both necessary and sufficient to drive Tfh differentiation (253–255). Not much is known about specific metabolic requirements of Tfh cells; however, recent evidence suggest that these cells have reduced mTORC1 activity and are less glycolytic compared with Th1 cells (256).

Th17 cells are characterized by production of the signature cytokines IL-17A, IL-17F, and IL-22 (257–261) and expression of IL-23R and the chemokine receptor CCR6 (262, 263). The transcription factor Rorγt is considered to be the master regulator of Th17 cells (264). A natural ligand for Rorγt – an intermediate of cholesterol biosynthesis pathway – has recently been identified (265, 266). The differentiation into Th17 cells requires TGF-β1 in the presence of proinflammatory cytokines, including IL-1β, and STAT3-activating cytokines, such as IL-6 or IL-21 (267), whereas IL-23 plays an essential role in sustaining Th17 differentiation and promoting their survival and acquisition of pathogenic effector potential (268–270). Similar to Th1 cells, Th17 cells require mTORC1 activation during differentiation, but are additionally dependent on HIF-1α activity and are thought to heavily rely on glycolysis, as blocking glycolysis with 2-deoxy-d-glucose (2-DG) inhibits Th17 cell differentiation (271, 272). Recently, fatty acid synthesis (FAS) was also shown to dictate the balance between Th17 and Treg differentiation, where *de novo* FAS promoted Th17 over Treg cell differentiation (273). Th17 cells are enriched at mucosal sites where they play a key role in protection against various extracellular pathogens, including fungal and bacterial infections (267, 274–276). However, Th17 cells have also been implicated in several chronic inflammatory and autoimmune disorders, including intestinal inflammation (267, 268, 277) and neurological disorders (278).

Commensal microbiota modulates abundance and function of intestinal Th cells, and this is well exemplified by the effect on Th17 cells. GF mice show reduced frequencies of intestinal Th17 cells, which can be restored upon colonization with the segmented filamentous bacteria (SFB), Gram-positive bacteria belonging to the *Clostridiales* genus (279, 280). How SFB promote effector T cell polarization and accumulation is still not completely understood, but it was proposed that SFB colonization increases levels of the acute-phase protein serum amyloid A (SAA), which conditioned intestinal MPs to promote Th17 cells (279). More recently, presentation of SFB antigens on MHC II molecules by intestinal DCs was implicated in SFB-specific Th17 cells differentiation (281, 282). Of note, it remains unknown whether equivalent single bacteria species able to promote Th17 cells exist in humans. Additionally, it was shown that Th17 cells could directly detect microbial-associated molecules through TLR2 and that this potentiated Th17 responses (283).

### Regulatory T Cells in the Intestinal Mucosa

In comparison to the systemic immune compartment, the intestinal mucosa is significantly enriched in regulatory T cells and their non-redundant role in controlling intestinal inflammation is well documented (284, 285). The majority of these cells are Foxp3^+^ Treg cells. Foxp3 is a master transcriptional regulator of Treg cells, necessary for their development and maintenance (286). Treg cells can be generated in the thymus during development (tTreg cells), or in the periphery from conventional naive CD4^+^ T cells (pTreg cells). Differentiation of tTreg cells requires a transient TCR stimulation with a particular strength of TCR signaling following recognition of self-peptides, which corresponds to a TCR avidity between the one that dictates positive selection and the one that leads to negative selection (287). Induction of pTreg cells from naive CD4^+^ T cells *in vivo* occurs when antigen is presented under subimmunogenic or non-inflammatory conditions, during chronic inflammation, and in the setting of a tolerogenic microenvironment, which includes the intestinal LP (288). Indeed, the gastrointestinal tract is a preferential site of pTreg cell conversion; however, whether pTreg and tTreg have overlapping or distinct functions in maintaining gut homeostasis is an ongoing question (211).

The mechanisms used by Treg cells to suppress deleterious inflammatory responses have been extensively studied. They include production of regulatory cytokines (IL-10, TGF-β1, and IL-35), suppression by metabolic disruption, direct modulation of DC function, and cytolysis (289). In the context of intestinal homeostasis, IL-10 and TGF-β1 are of particular importance in enforcing tolerance and genetic deletion of IL-10, IL-10R, or impairment of the pathway results in microbiota driven intestinal inflammation in mice and humans (284, 285, 290).

Treg cells display specific adaptations that are tailored to the environmental context in which they operate. It was recently proposed that circulating Treg cells could be therefore divided into central, effector, and tissue-resident Treg cell populations (291). The existence of memory Treg cells has also been postulated (292–295). Tissue-resident Treg cells are long-term residents within various non-lymphoid organs, to which they adapt through transcriptional and metabolic reprograming (296). Potentially, each organ might have its own specific Treg cell population, and tissue-resident Treg cells have been described in skin, muscle, adipose tissue, placenta, and the intestine (296). Gut-resident Treg cells express chemokine receptors and adhesion molecules that facilitate gut homing, such as β7 family integrins and CCR9, expression of which is promoted by RA (121, 297), and G protein-coupled receptor 15 (GPR15) (298). Additionally, gut-resident Treg cells can be characterized by expression of the high affinity IL-2 receptor and the short-chain fatty acid (SCFA) receptor GPR43 (299). It is important to note that due to difficulties in distinguishing between effector and tissue-resident Treg cell populations the degree of plasticity between them remains unclear. Overall, Treg cells are thought to metabolically resemble memory T cells, in that they preferentially rely on lipid instead of glucose metabolism for energy generation; however, it remains largely unexplored whether particular subpopulations of Treg cells are metabolically distinct (220).

Commensals influence Foxp3^+^ Treg cell induction in the gut in several ways. For instance, the capsular polysaccharide A (PSA) of the Gram-negative anaerobic commensal *Bacteroides fragilis* was shown to promote IL-10 producing Foxp3^+^ Treg cells (300) through direct interaction of PSA with TLR2 on T lymphocytes (301). Furthermore, metabolites, such as SCFA, are emerging as key homeostatic signals provided by commensal microbiota to regulate local Treg cells. SCFA, such as butyrate and acetate, can act directly on mucosal Treg cells to promote their expansion. Mechanistically, butyrate appeared to promote pTreg cell induction by inhibiting histone deacetylases (HDAC). Butyrate-treated naive CD4^+^ T cells exhibited increased acetylation of the *Foxp3* locus, including the key CNS1 enhancer region that is essential for pTreg differentiation (299, 302, 303). Of note, recent reports indicate that pTreg cells induced by the commensal microbiota antigens can be distinguished from other pTreg cells based on their expression of Rorγt (304, 305), suggesting an additional level of Treg cell specialization may exist in the gut.

### Autophagy Regulates T Cell Survival

The first indication of the importance of the autophagy pathway for T lymphocyte homeostasis *in vivo* came from the study of *Atg5*^−/−^ fetal liver chimeric mice where decreased numbers of thymic and splenic autophagy-deficient CD4^+^ and CD8^+^ T cells were reported (306). Several genetic models were subsequently employed to investigate a specific role for autophagy in T cell development. These studies investigated T cells within the thymus and in the secondary lymphoid organs, including spleen and lymph nodes. T cell-specific deletion of *Atg3, Atg5, Atg7, Atg16l1, Vps34*, or *Beclin1* consistently showed decreased frequencies and numbers of CD4^+^ and CD8^+^ T in the secondary lymphoid organs, whereas thymic development was largely unperturbed (200, 307–312). However, the requirement for autophagy in thymic development seems to be dependent on the system that was used to generate T cell-specific deletion of autophagy genes. While mice where Cre recombinase expression was driven by the Lck promoter (Cre expression occurs at the double negative stage) showed a mild, but significant, reduction in thymocyte numbers (308, 313), mice where the CD4 promoter was used to drive Cre expression (resulting in excision during the later double positive stage) did not show any significant changes in thymocyte development (200, 307, 310, 312). One exception is NKT cells, which require autophagy during thymic development (309, 314, 315).

These studies also observed increased proportions of effector/memory phenotype (CD62^low^ CD44^hi^) T cells among peripheral autophagy-deficient T cells, which was interpreted to be a result of decreased survival of naive T cells (308, 311). However, this phenotype might also occur as a result of lymphopaenia-induced proliferation (200). The role of autophagy in activated T cells has been predominantly studied *in vitro*. The question of when autophagy is activated in T cells remains controversial. While autophagy is known to be negatively regulated by mTORC1 signaling in many cell types, and therefore inversely correlates with cell proliferation (21), early *in vitro* studies indicated that TCR triggering induces autophagy in T lymphocytes and reported that chemical or genetic blockage of the autophagy pathway impaired T cell activation and proliferation (306, 308, 316, 317). Autophagy-deficient T cells also showed increased apoptosis during prolonged *in vitro* culture (308) or after activation (310, 312). Some of the studies also observed decreased production of effector cytokines by *in vitro* activated autophagy-deficient T cells, including IL-2, IL-17A, and IFNγ (309, 317). Conversely, another study reported increased IL-2 production by *Atg7*-deficient CD4^+^ T cells after TCR cross-linking (318). In several studies, these defects in autophagy-deficient T cells were linked to impaired organelle homeostasis, particularly mitochondria homeostasis, and were associated with an increase in ROS production (307, 308, 311). Increases in ER mass and changes in intracellular calcium signaling were also implicated in this impaired fitness and survival (318). However, whether this is indeed the mechanistic explanation for the decreased fitness of autophagy-deficient CD4^+^ T cells *in vivo* remains unclear, as other studies did not observe any increase in mitochondrial mass or ROS production (310, 319), and mitochondria were shown to be excluded from autophagosome degradation in activated wild-type CD4^+^ T cells (317). Further complication arises from the findings that although increased ROS can be detrimental for T cells (320), ROS production is increased after TCR triggering and is, in fact, required for T cell proliferation, particularly in CD8^+^ T cells (321–324). It was also proposed that imbalanced expression or accumulation of apoptosis-related proteins might contribute to the defective proliferation and survival of autophagy-deficient T cells. However, these results are difficult to interpret as increased levels of both proapoptotic (308–310) and anti-apoptotic proteins (Bcl-2, Mcl-1) (308–310) have been reported in autophagy-deficient T cells. Analysis of mice with T cell-specific deletion of *Vps34* suggested that decreased levels of IL-7Rα expression on T cells might be involved, although this was not attributed to the cell-intrinsic effects of autophagy deficiency (307, 319). Treatment with the pan-caspase inhibitor zVAD could partially rescue the apoptotic phenotype of autophagy-deficient T cells (308, 310); however, it did not rescue the defects in T cell proliferation (309). Furthermore, it is worth pointing out that in addition to its role in T cell survival and proliferation, autophagy has also been reported to promote cell death in activated T cells under some circumstances (316, 325, 326).

Interestingly, autophagy has been directly linked to regulation of signaling cascades downstream of the TCR. For example, in activated, but not in naive T cells, autophagy was shown to selectively target Bcl-10 for degradation in a p62-dependent manner, limiting NFκB-dependent effector responses, including IL-2 secretion (29). NFκB signaling plays an important role in many aspects of activated T cell physiology, including entry into cell cycle (327), but the strength of NFκB signaling can also influence differentiation into distinct Th cell subsets; therefore, autophagy might contribute to these processes by regulating the NFκB pathway in activated T cells.

Recent reports identified a role of autophagy in the formation of memory CD8^+^ T cells during viral infections (199, 200, 328). These studies, which focused on *in vivo* responses of autophagy-deficient CD8^+^ T cells to influenza or lymphocytic choriomeningitis virus infections, revealed new aspects of autophagic regulation of T cells. Although distinct genetic approaches were used to generate mice with a selective autophagy deficiency in T cells, all observed that autophagy was dispensable during the early expansion phase of antigen-activated CD8^+^ T cells during viral infection (199, 200, 328). In addition, autophagy-deficient T cells did not show defects in effector cytokine production and were capable of controlling virus titers during the early phases of infection (199). However, activation of the autophagy pathway was shown to be crucial during the transition phase between late effector to memory T cells and mice with autophagy-deficient CD8^+^ T cells did not mount proper memory CD8^+^ T cell responses during secondary challenge (199, 200, 328). While the mechanism behind the requirement for autophagy in memory CD8^+^ T cell responses remains to be elucidated, comparison of metabolic profiles between WT and *Atg7*-deficient memory CD8^+^ T cells suggested that metabolic adaptation might be involved (199). In addition, CD8^+^ T cells from aged mice (200) and senescent human CD8^+^ T cells exhibited low levels of autophagy, which in human senescent cells was associated with high p38 kinase activity (329). Importantly, increasing autophagy levels was shown to boost memory CD8^+^ T cell responses after influenza vaccination in aged mice (200). Of note, it remains unknown whether autophagy is equally essential for the development of CD4^+^ T cell memory cells and whether autophagy plays any role in the formation of T_RM_ cells, including mucosal T_RM_ cells.

Overall, while it is clear that autophagy plays a crucial role in the maintenance of peripheral CD4^+^ and CD8^+^ T cells and memory CD8^+^ T cells (Figure 3), it is still not completely understood how autophagy influences different aspects of T cell physiology in naive and activated T cells. Methodological difficulties of monitoring autophagy, differences in the genetic models used, as well as differences between *in vivo* and *in vitro* stimuli might underlie some of the discrepancies observed. Indeed, autophagy is an essential homeostatic process for all eukaryotic cells, including T cells. Therefore, genetic deletion of essential autophagy genes early during T cell development, using CD4 or Lck promoter driven Cre-lox technology, can mediate major changes in autophagy-deficient T cells, including global alterations in gene expression (313). Arguably, this makes it difficult to study the function of essential autophagy genes in isolation on a particular process or step of T cell development. In order to avoid this complication, several groups attempted to use an inducible system to selectively knock-out autophagy genes at a chosen time point (311, 328), or used the granzyme B promoter to selectively drive Cre expression in mature CD8^+^ T cells (199). However, in the context of intestinal homeostasis, it is important to remember that in terms of revealing how polymorphisms in autophagy genes are linked to disease susceptibility, the system where autophagy is perturbed from the beginning of T cell development may be physiologically relevant.

### Autophagy Regulates the Balance of Intestinal CD4^+^ T Cell Subsets

**Figure 5:**
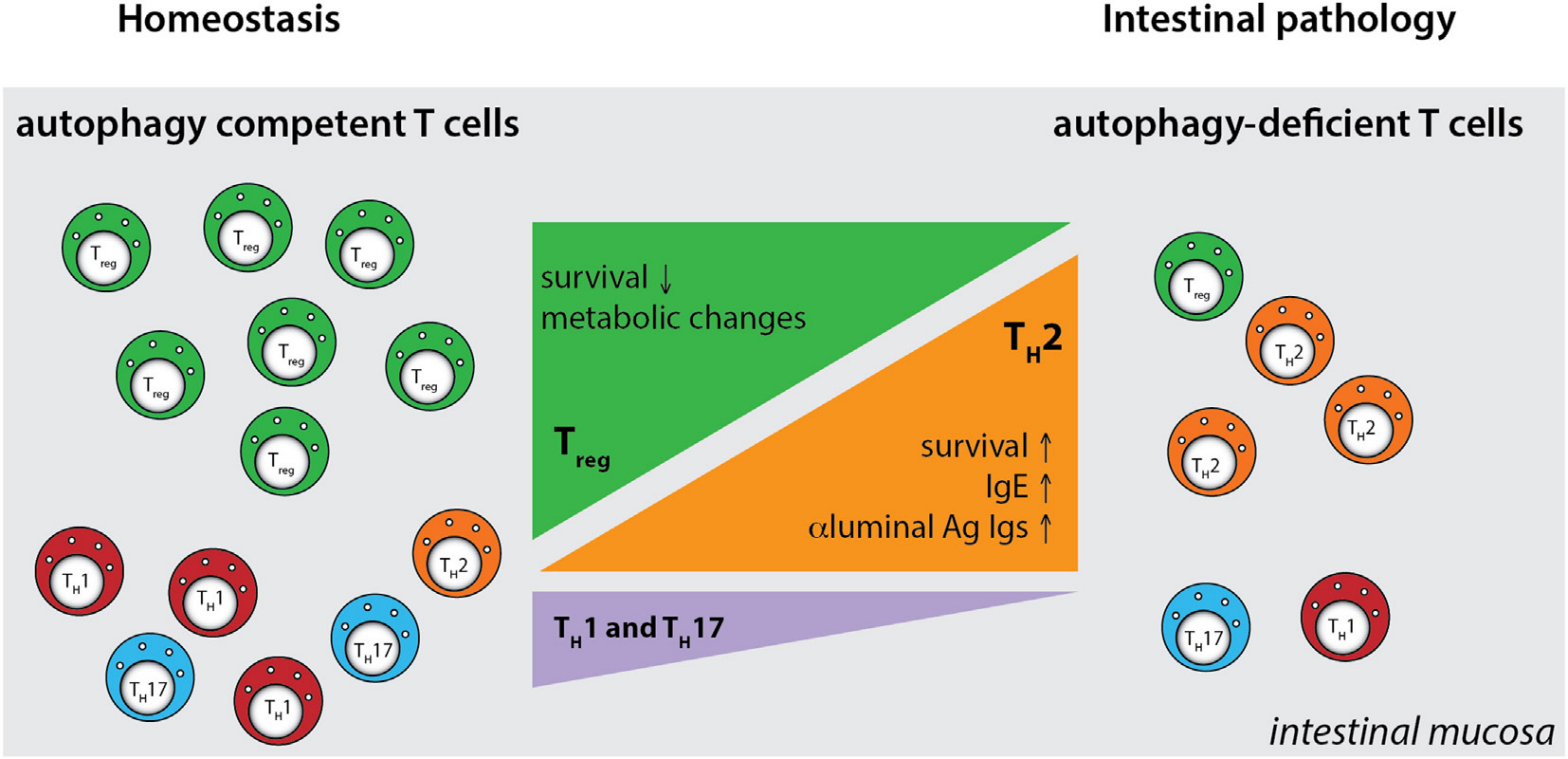
**Defective autophagy alters the balance of the intestinal CD4^+^ T cell subsets**. T cell-specific ablation of autophagy results in a strong decrease in the numbers of Treg cells and also reductions in Th1 and Th17 effector cells. Conversely, loss of autophagy promotes expansion of intestinal Th2 cells, leading to aberrant responses to luminal antigens and subsequent intestinal pathology. Ag - antigen.

Although these studies investigated the role of several autophagy genes in T cell physiology, intestinal populations of T cells were not analyzed. We studied the role of the IBD susceptibility gene *Atg16l1* in the intestinal T cell homeostasis and function (312). This study showed that autophagy pathway was essential for the maintenance of T cells in the small intestinal and colonic mucosa, as CD4^+^ and CD8^+^ T cell numbers were strongly reduced in the mice with selective Atg16l1-deficiency in the T cell compartment (312). Moreover, detailed examination of intestinal CD4^+^ T cell subsets revealed that Atg16l1-deficiency differentially affected distinct subsets of CD4^+^ T cells in the gut; it strongly compromised the Treg cell compartment, whereas the Th2 population was selectively enhanced. In addition, Th1 and Th17 cells, which are commonly implicated in intestinal pathology, were reduced in the gut of mice with *Atg16l1*-deficient T cells (Figure 5). These changes were most pronounced in the intestinal mucosa, suggesting different requirements for autophagy for the accumulation and survival of distinct subsets of CD4^+^ T cells within the intestinal environment. Aged mice with T cell-specific deletion of *Atg16l1* developed spontaneous and progressive intestinal pathology, which was preceded by the production of Th2-associated antibodies toward dietary and commensal antigens (312). These aberrant type 2 responses resulted not only from a lack of sufficient Treg control but also from cell-intrinsic dysregulation, as T cell-intrinsic autophagy limited the expansion of Th2 cells (312).

**Figure 6:**
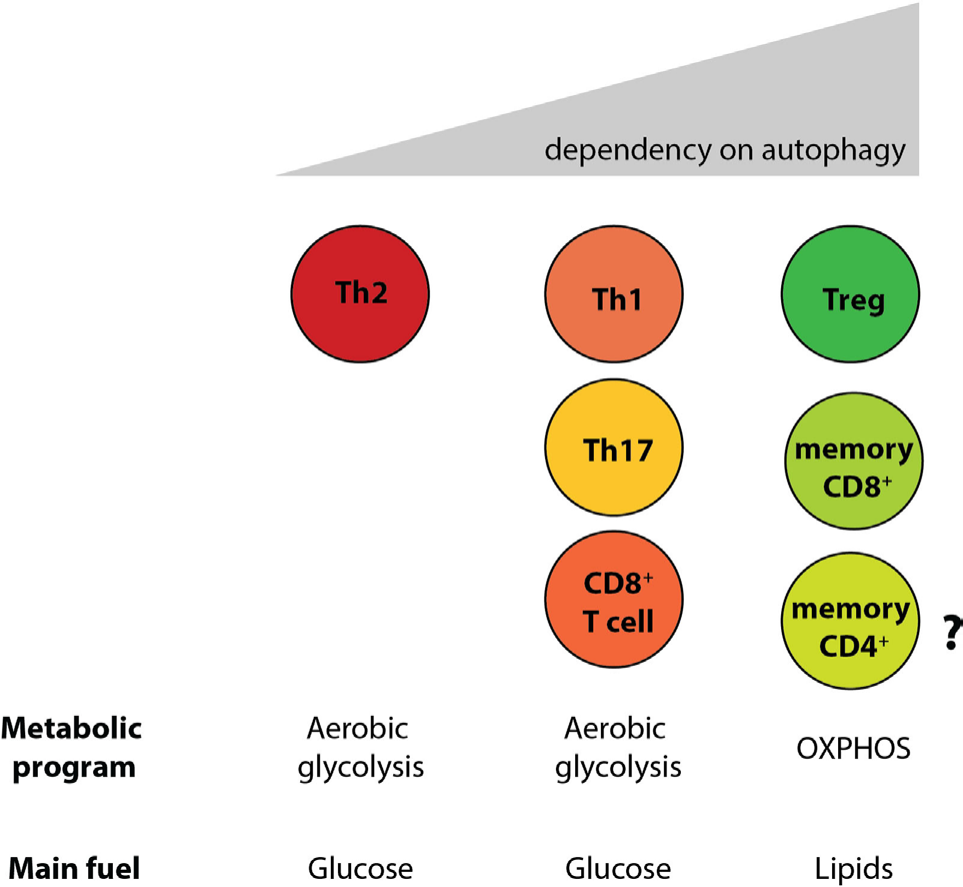
**Differential reliance on autophagy within intestinal T cell subsets corresponds to their different metabolic profiles**. Cell-intrinsic defects in autophagy negatively affect T cell subsets, with the exception of Th2 cells. Treg cells and memory CD8^+^ T cells, which rely on lipid metabolism for survival, were shown to be the most sensitive to autophagy perturbation. However, the role of autophagy CD4^+^ memory T cells formation has not yet been investigated.

By generating mice with a Treg cell-specific deletion of *Atg16l1*, we demonstrated that cell-intrinsic autophagy is indispensable for Treg cell maintenance in the periphery and thus for the control of effector T cell responses, as these mice developed severe systemic and gastrointestinal inflammation. These findings are consistent with an independent study, which reported that Treg cell-specific deletion of *Atg5* or *Atg7* led to the spontaneous development of severe multi-organ inflammation (330). Autophagy-deficient Treg cells exhibited marked phenotypical changes, including increased cell cycling, production of Th effector cytokines, and reduced Foxp3 expression, that were associated with increased activation of mTORC1 (312, 330). In addition, autophagy-deficient Treg cells showed a distinct metabolic profile, with increased glycolysis and reduced expression of genes involved in FAS/FAO (312, 330). Increased glycolysis could be a more general phenomenon observed in autophagy-deficient T cells, as others reported a similar glycolytic shift in autophagy-deficient CD8^+^ memory T cells (200). Based on recent evidence that metabolic changes are emerging as an important part of tissue-specific reprograming of tissue-resident Treg cells (291, 296), we predict that these metabolic perturbations introduced by autophagy deficiency strongly affect intestinal Treg cells, which rely on fatty acid metabolism, similar to memory T cells (Figure 6) (312). In contrast, Th2 cells seem resistant to these metabolic changes, presumably due to their ability to cope with prolonged high levels of glycolysis, perhaps explaining why this particular Th cell subset were not negatively affected by autophagy deficiency (312) (Figure 6).

In summary, autophagy differentially regulates survival of Treg cells and Th2 cells; T cell-intrinsic autophagy might facilitate metabolic adaptations that are required for the survival of intestinal Treg cells, while Th2 cell are resistant to metabolic perturbations introduced by autophagy deficiency. As polymorphisms in *ATG16L1* and other autophagy genes have been linked to IBD susceptibility, these results identify a potential novel mechanism that links genetic susceptibility in the autophagy pathway to intestinal inflammation through dysregulation of mucosal T cell responses. Moreover, as defective Treg and increased Th2 responses at the mucosa are observed in food allergies and asthma, and since polymorphisms in the essential autophagy gene *Atg5* have been implicated in asthma susceptibility (331, 332), these findings might also have implications for these conditions that affect mucosal tissues.

## Conclusion and Perspectives

In recent years, researchers have employed the Cre-lox system to generate transgenic mice with conditional knock-out of autophagy genes to study the role of autophagy in a cell type-specific manner. While this is undoubtedly a powerful tool to look at autophagy functions *in vivo*, it is important to remember that these are rarely perfect, as few promoters show complete specificity for one cell type and off-target effects of autophagy gene deletion should be considered. Nevertheless, studies using distinct Cre lines, together with those using human cells with disease-associated autophagy mutations, have identified several key mechanisms through which autophagy can influence the functions of distinct cell populations in the gut (Table 1). However, some observations remain inconclusive and the molecular mechanisms through which autophagy controls such a range of functions in diverse cell types are not well defined (Table 1). It is important to point out that autophagy is a fundamental process in eukaryotic cells and complete inhibition or deletion of essential autophagy genes often results in strong perturbations in cellular physiology, including activation of compensatory or rescue pathways. Future studies may employ more refined approaches, such as using knock-in mice harboring disease-associated autophagy gene alleles.

**Table 1:** **Consequences of autophagy deficiency in the intestine**.

Cell type	Genetic manipulation	Phenotype/results	Cautionary notes
Intestinal epithelium	*Atg5*^Δvillin^ *Atg16l1*^Δvillin^	Decreased defense against intestinal intracellular bacteria (110, 111)	
	*Atg16l1^HM^* *Atg5*^Δvillin^ *Atg16l1^T316^*^A^ *CD patients with ATG16L1^T300^*^A^	Defects in structure and secretory functions in Paneth cells and goblet cells; increased susceptibility to DSS-induced colitis (13, 114, 115, 118)	In the mouse models, the phenotype may depend on the presence of norovirus infection, or additional perturbations in compensatory pathways like the ER stress pathway (12, 116)

Mononuclear phagocytes	*Atg5*^ΔCD11c^ *CD patients with ATG16L1^T300^*^A^	Decreased MHC II antigen presentation on DCs (137, 148)	Relevance for intestinal DC function *in vivo* remains to be established
	*Atg16l1^T316^*^A^ *CD patients with ATG16L1^T300^*^A^	Decreased defense against intestinal intracellular bacteria (12, 148)	
	*Atg16l1*^−/−^ fetal liver chimeric mice *Map1lc3b^−/−^* *CD patients with ATG16L1^T300^*^A^	Defective inflammatory cytokine regulation: impaired autophagy results in increased IL-1β production (155, 156) and in increased susceptibility to DSS-induced colitis and sepsis (155, 158)	Autophagy seems to play a dual role in regulating some inflammatory cytokine pathways, as it was shown to both limit (158, 168, 169) and facilitate inflammasome and type I IFN pathways (147, 165, 167) – it remains unclear which effect dominates in intestinal mucosal MP

B cells	*Atg5*^ΔCD19^ *Atg7*^ΔCD19^	Impaired survival of plasma cells and memory B cells; decreased secondary antibody responses after influenza infection (195, 197, 198)	Effects on local mucosal antibody responses not yet investigated
			Effects of autophagy deficiency on primary antibody production remains unclear, as both decreased and increased serum antibody levels have been reported (195, 196)

T cells	*Atg5*^−/−^ fetal liver chimeric mice *Atg7*^ΔLck^ *Atg16l1*^ΔCD4^	Decreased survival of peripheral CD4^+^ and CD8^+^ T cells, including intestinal lamina propria T cells (306, 308, 312)	
	*Atg7*^ΔCD4^ *Atg7*^ΔGzmb^ *Atg5*^ΔGzmb^	Defects in the formation of memory CD8^+^ T cells and secondary responses to viral infections (199, 200)	The requirements for autophagy for CD4^+^ memory T cells or intestinal T_RM_ remain to be determined
	*Atg16l1*^ΔCD4^ *Atg16l1*^ΔFoxp3^ *Atg5*^ΔFoxp3^ *Atg7*^ΔFoxp3^	Compromised Treg cell number in the intestine; increased in Th2 type responses, spontaneous intestinal pathology (312, 330)	

*Δ designates promoter that is used to drive the Cre expression in the transgenic mouse strain*.

*CD, Crohn’s disease; DCs, dendritic cells; DSS, dextran sulfate sodium; ER, endoplasmic reticulum; Gzmb, granzyme B; HM, hypomorphic; IFN, interferon; Lck, lymphocytes protein tyrosine kinase; MP, mononuclear phagocytes; T_RM_, tissue-resident memory T cells*.

The role of autophagy has been investigated for the main leukocyte subsets and we know a lot about how it regulates different aspects of their differentiation and function. However, the majority of these observations were made using leukocytes derived from secondary lymphoid tissues and extrapolation to the intestinal mucosa should be treated cautiously, as mucosal immune cells often display very distinct properties from their systemic counterparts. A good example of this is our discovery that enhanced Th2 responses in autophagy-deficient CD4^+^ T cells occur primarily within the intestinal mucosa. Emerging literature indicates that effector and memory T cell responses in tissues are dependent on metabolic adaptations that allow T cells to survive and function in environments with altered availability of nutrients and growth factors. We speculate that autophagy plays a key role in endowing T cells with the metabolic flexibility to adapt these challenges. Thus, the immune manifestations of autophagy deficiency depend not only on the cell type considered but also on the tissue context.

Many important issues still remain to be addressed to give a more complete picture of how autophagy regulates intestinal immune homeostasis. These include a comprehensive analysis of the effects of autophagy on unique populations of innate leukocytes that are present in the gut, such as ILCs and IELs. In addition, the influence of autophagy on CD4^+^ T cell memory formation and development of T_RM_ cell responses in the mucosa has still to be determined. An improved understanding of the molecular mechanisms that make Th2 cells resistant to the effects of autophagy deficiency is also required.

Autophagy is an attractive therapeutic target and several autophagy modulating compounds are already in clinical trials for the treatment of various disorders (333). For instance, the autophagy-enhancing drug carbamazepine has been shown to ameliorate hepatic fibrosis in the mouse model of α1-antitrypsin deficiency liver cirrhosis, and this drug is currently in phase II of clinical trials (333, 334). Additionally, an autophagy-inducing agent has been shown to decrease pathology in a mouse model of chemically induced colitis (335, 336). The challenge, however, is to identify agents that can specifically induce autophagy with minimal side effects on other cellular processes. Indeed, considerable effort in the field of autophagy research is currently focused on finding small molecules that can induce autophagy in a very selective manner (337, 338). Interestingly, as some of the dietary-derived compounds, including RA (339) and vitamin D (340), have been shown to enhance autophagy, it is tempting to speculate that the use of such natural inducers could prove beneficial for treatment of intestinal inflammatory disorders.

## Author Contributions

AK, JP, and KM prepared the manuscript text. AK and JP preparedthe figures.

## Conflict of Interest Statement

The authors declare that the research was conducted in the absence of any commercial or financial relationships that could be construed as a potential conflict of interest.
